# Early Markers of Sickle Nephropathy in Children With Sickle Cell Anemia Are Associated With Red Cell Cation Transport Activity

**DOI:** 10.1097/HS9.0000000000000002

**Published:** 2017-12-20

**Authors:** John Brewin, Sanjay Tewari, Anke Hannemann, Halima Al Balushi, Claire Sharpe, John S. Gibson, David C. Rees

**Affiliations:** 1 Red Cell Biology, King's College London, London, UK; 2 Department of Haematology, King's College Hospital, London, UK; 3 Department of Veterinary Medicine, University of Cambridge, Cambridge, UK; 4 Department of Renal Sciences, King's College London, London, UK

**Keywords:** hyperfiltration, K^+^ permeability, proteinuria, sickle cell disease, sickle cell nephropathy

## Abstract

The early stages of sickle cell nephropathy (SCN) manifest in children with sickle cell anemia (SCA) as hyperfiltration and proteinuria. The physiological conditions of the renovascular system are among the most conducive to hemoglobin S polymerization in the body and will magnify small changes in red cell volume thus crucially modulating intracellular concentrations of hemoglobin S. This large cross-sectional study of children with sickle cell anemia measured glomerular filtration rates and microalbuminuria to report prevalence, clinical correlates and uniquely, association with key red cell cation transport mechanisms. One hundred and twelve patients (mean age 10.7 ± 4.1) were recruited. The prevalence of hyperfiltration and microalbuminuria was 98% and 15.1%, respectively. Glomerular filtration rates did not vary with age, but proteinuria became more prevalent with increasing age. Both features associated with markers of hemolysis, while elevated hemoglobin F was protective, but no association was seen with systolic or diastolic blood pressure. In multivariate analysis, both Gardos channel (β = 0.476, *P* < 0.001) and KCl co-transporter (KCC; β = −0.216, *P* = 0.009) activity, alongside age (β = 0.237, *P* = 0.004), remained independently predictive for microalbuminuria. Increased activity of Gardos channel and P_sickle_ positively associated with microalbuminuria, while increased KCC activity associated with a reduction in microalbuminuria. This study demonstrates a direct link between the abnormally active red cell cation transport systems in sickle cell disease and sickle organopathy. Small variations in the activity of these transport mechanisms predict for SCN and measurement of them may help identify those at risk, while pharmaceutical manipulation of these excessively active systems may ameliorate their risk.

## Introduction

Sickle cell anemia (SCA) is one of the most common monogenic disorders in the world. It arises from a single point mutation (c.A20T, p.Glu7Val), in the β globin gene, which causes hemoglobin polymerization and abnormal red cell conformations, leading to vessel occlusion and ischemia-reperfusion injury. The disease is characterized by severe episodes of pain and dysfunction of virtually every organ system in the body. However, there is significant variation between patients in the severity of this condition.^1^ As yet, it is not possible to reliably identify those individuals most at risk of developing the more severe complications of SCA, including renal damage.

Progressive renal damage, termed sickle cell nephropathy (SCN) occurs in up to one-third of all patients and is strongly associated with increased mortality.^2^ Clinical manifestations begin early in childhood with glomerular hyperfiltration, hyposthenuria, and distal renal tubular acidosis initially, and albuminuria developing subsequently. The incidence of hyperfiltration and albuminuria in children has been previously reported as 76%, and 15.9%, respectively.^3^ Hyperfiltration can develop as early as 12 months of age with an age-dependent increase until the second decade of life, whereas albuminuria generally only develops in the second decade of life.^3–6^ A significant number of such patients go on to develop renal failure as adults, requiring dialysis or transplantation.^7^ Patients with SCA have a lowered life expectancy but while more sophisticated medical provision has extended their lifespan, the proportion of patients progressing to chronic organ damage including SCN has consequently increased.^8^

Although the full pathophysiology of SCN is incompletely understood, the kidney microenvironment presents conditions favorable toward sickle hemoglobin polymerization. Such polymerization is influenced by the local oxygen tension and promoted by both acidosis, which decreases the oxygen affinity of hemoglobin S (HbS), and hypertonicity, which encourages erythrocyte dehydration by osmosis, thereby increasing red blood cell (RBC) HbS concentration. The importance of RBC HbS concentration in the development of HbS fibers has long been recognized.^9^ As deoxygenated RBCs squeeze through the microvasculature, there is a lag time before the formation of HbS fiber polymers. Most of the time, the red cells escape the hypoxic microcirculation before this point and do not trigger sickling of the red cell. However, the delay time to polymerization is highly dependent on intracellular HbS concentration. This lag time is inversely proportional to a high power of [HbS] meaning a little solute loss and dehydration resulting in a small rise in [HbS] markedly encourages sickling. Within the kidney, high oxygen consumption leads to increased hypoxemia. The blood is particularly acidic and hypertonic and the blood flow is slowed as it passes through the medullary vasa recta. Together, these factors contribute to a shorter HbS polymerization lag time, and a longer period within the microvasculature in the kidney.

Given the importance of solute loss and red cell dehydration, it is clear HbS polymerization will be heavily influenced by the activity of cation transport of the red blood cell membrane. Sickle RBCs have unusually high permeability to cations, compared to normal RBCs. Three transport mechanisms are primarily responsible for this aberrant state.^10^ They are the KCl co-transporter (KCC), which mediates obligatory coupled K^+^ and Cl^−^ efflux; an ill-defined cation conductance, sometimes referred to as P_sickle_, which is activated by deoxygenation, HbS polymerization and red cell shape change; and the Gardos channel, a Ca^2+^-activated K^+^ conductance, stimulated in particular by Ca^2+^ entry via P_sickle_. Solute loss via these transport systems causes RBC dehydration and elevation of intracellular [HbS] leading to a greatly increased propensity to polymerize with a shorter lag time.

Small, inherited variation in the activity of these transporters would cause similar variation in an individual's HbS polymerization lag time and thus propensity to microvascular occlusion and tissue damage. We predict that this variation would be most marked in the renal system given the unique conditions that the red blood cells are exposed to as they pass through the medullary vasculature. We therefore investigated the hypothesis that children with increased red cell cation transport activity would be predisposed toward early renal damage, in the form of hyperfiltration and microalbuminuria.

## Methods

### Patients

One hundred and twelve children (>4 years old) with SCA (HbSS) attending the Pediatric Hematology clinic at King's College Hospital, London, UK, were recruited for the study. Patients transfused in the preceding 4 months or taking medications known to alter RBC permeability (eg, dipyridamole and Ca^2+^ channel blockers) were excluded, but the study included those on hydroxyurea (HU). All patients were in the steady state, and had been without acute symptoms for at least 7 days. Standard laboratory parameters, together with age, height, weight, and blood pressure were recorded. GFR was calculated using the Schwarz method if age ≤17^11^ and MDRD, allowing adjustment for ethnicity, if >17 years of age.^12^ Both systolic and diastolic blood pressure recordings were compared with the reference ranges established for a pediatric cohort with SCA^13^ and categorized as normo- or hypertensive for both measurements.

### Laboratory assays

RBC samples were washed in simple 3-(N-morpholino) propanesulfonic acid (MOPS)-buffered saline, comprising (in mM): 140 NaCl, 5 KCl, 1.1 CaCl_2_, 10 MOPS, 5 glucose, pH 7.4 at 37°C. Oxygen tension was controlled using a Wösthoff gas mixing pump with RBCs incubated in Eschweiler tonometers. RBC permeability was assessed using radioactive tracers (^86^Rb^+^) to measure activity of the main cation transport systems involved in RBC dehydration: KCC, Gardos channel and P_sickle_. KCC was measured as Cl^−^-dependent K^+^ transport using NO_3_^−^ to substitute for Cl^−^. The Gardos channel was measured as clotrimazole-sensitive K^+^ transport. P_sickle_ is defined as the deoxygenation-induced Cl^−^-insensitive K^+^ transport in the continued presence of clotrimazole. This method separates Gardos channel activity from that of P_sickle_. Assays were carried out in the presence of ouabain and bumetanide to exclude any contribution of flux via the other 2 main RBC cation transporters, the Na^+^/K^+^ pump and the Na^+^-K^+^-2Cl^−^ cotransporter. The concentration for the respective inhibitors was as follows: 5 μM for clotrimazole, 10 μM for bumetanide, and 100 μM for ouabain. For full details of methods, see Hannemann et al.^14^

### Statistical analysis

Statistics were performed using IBM SPSS, New York, USA. Variables were approximated to normal distribution using logarithmic transformation if necessary. Simple linear regressions were performed for each parameter to search for potential correlation with estimated glomerular filtration rate (eGFR) and albumin/creatinine ratio (ACR). For binary variables, independent *t* tests were calculated. Multiple linear regressions were used to explore models that better predicted each outcome variable. All variables that were significantly correlated with the outcome of interest (*P* < 0.05), were considered in each regression. Models were built with a forward stepwise approach. The final models included the variables that remained significantly associated with eGFR or ACR after adjustment for the other variables in the models. R-squares (R^2^) were used as measures of variance explained by the models.

## Results

A total of 112 patients were recruited to the study. All children had HbSS genotype. The mean age was 10.7 ± 4.1 years (range 4–19 years). There was an even split of gender within the cohort. The clinical profile of the patients is summarized in Table 1.

**Table 1:**
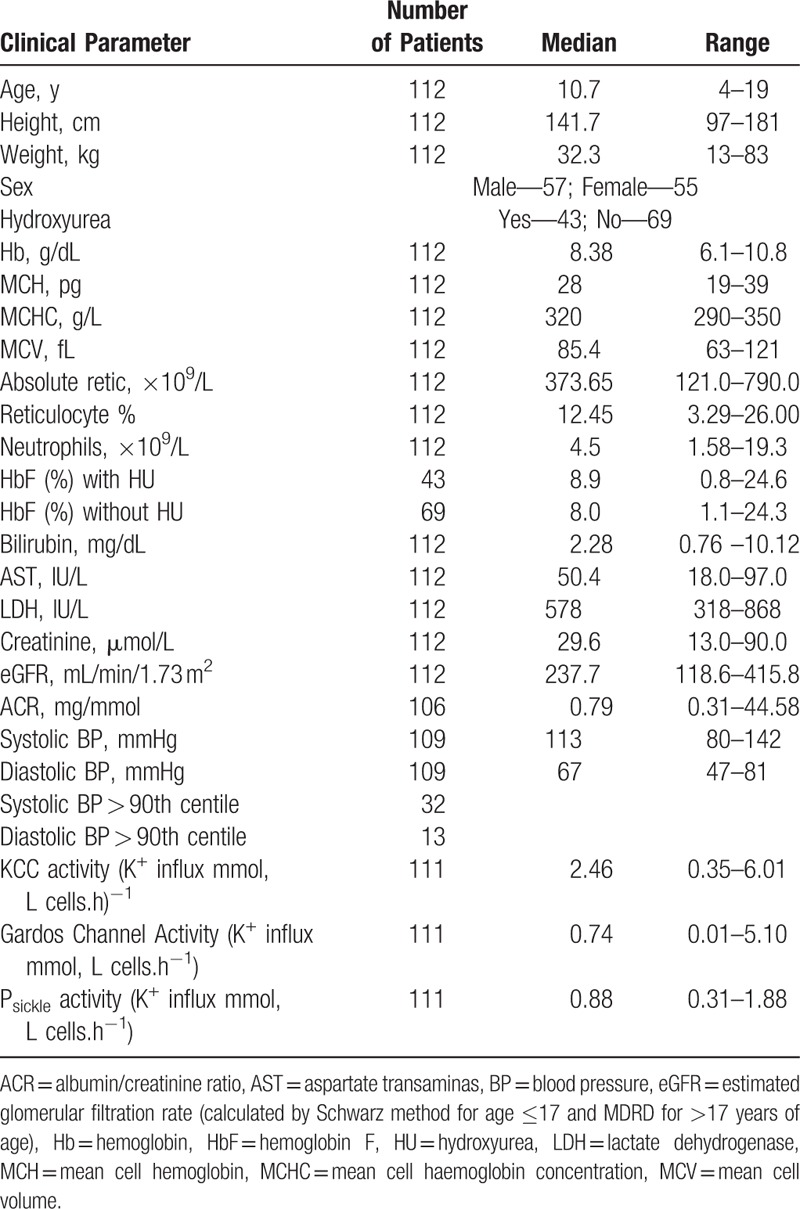
Clinical Profile of Patients

**Figure 1:**
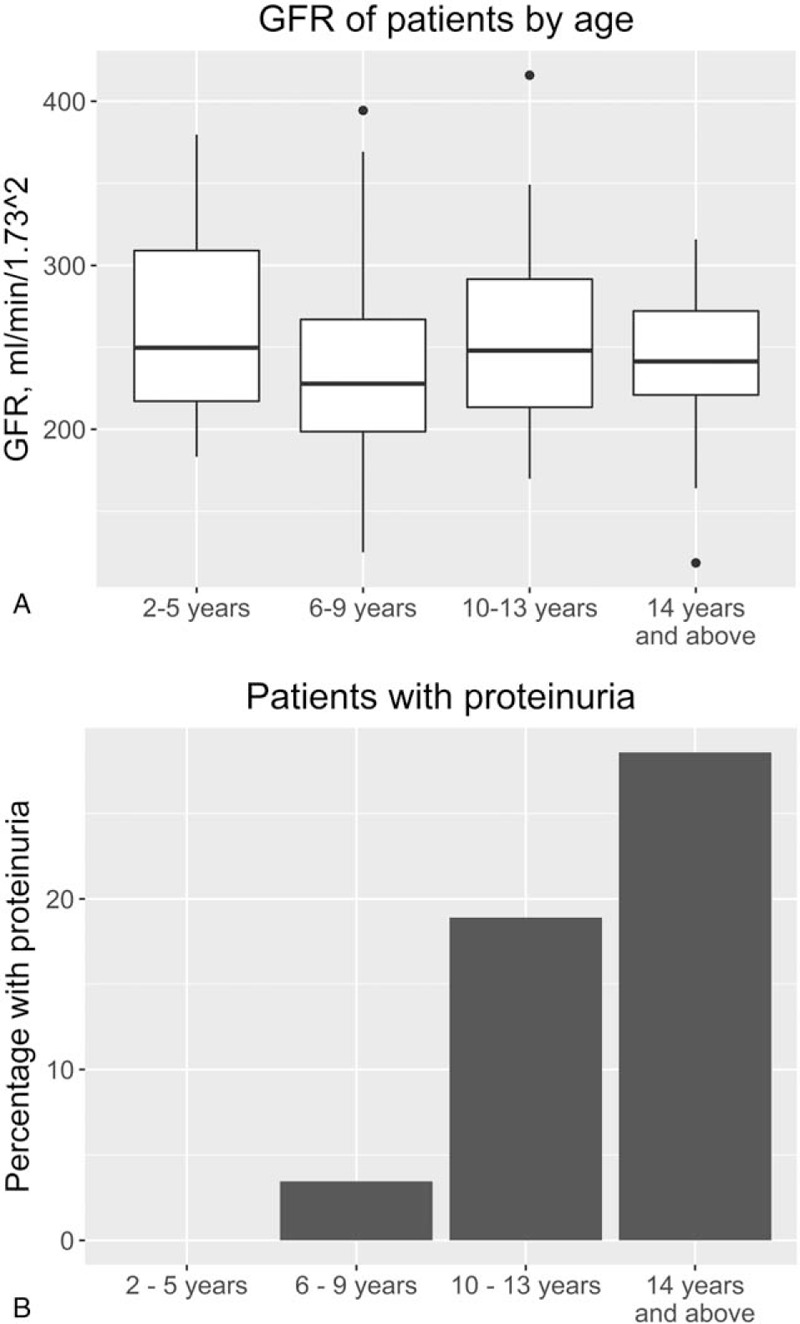
(A) Estimated glomerular filtration rate (eGFR) measurements by subset of age. (B) Percentage prevalence of proteinuria, defined as urinary albumin/creatinine ratio >3 mg/mmol, by subset of age. Age categories: 2–5 years (n = 22), 6–9 years (n = 29), 10–14 years (n = 37), >14 years (n = 28).

The exact definition of glomerular hyperfiltration is not well established and ranges from 125 to 175 mL/min/1.73 m^2^.^15^ Age and ethnicity are important factors in determining an appropriate threshold. Pediatric studies have demonstrated thresholds between 130 and 140 mL/min/1.73 m^2^ are most commonly used,^16^ while another retrospective review established 135 mL/min/1.73 m^2^ was appropriate in all children.^17^ A study looking at African American adults determined hyperfiltration as a GFR > 140 mL/min/1.73 m^2^.^18^ We therefore chose this higher threshold for our current study. Hyperfiltration was observed in 109 out of 112 patients (98%). The mean eGFR was 249 ± 56 mL/min/1.73 m^2^ (range 118.6–415.8). There was no significant change in the eGFR measurements with age (Fig. 1A). Urinary ACR was measured in 106 patients. Microalbuminuria defined as an ACR >3 mg/mmol is established in adult populations and this is commonly used in the pediatric population too.^19^ A large US-based study^20^ confirmed the appropriateness of this threshold. By this definition, 16 had proteinuria (15.1%). The age range was 9 to 19 years and the prevalence increased with age (Fig. 1B). In patients over the age of 14 years, the prevalence was 28.5%.

Correlations of eGFR and ACR with the other clinical measurements taken at the same time were assessed (Table 2). In univariate analysis, statistically significant associations between eGFR and markers of hemolysis were seen. Specifically, there was an inverse relationship with steady state hemoglobin (Hb) (r = −0.34, *P* < 0.001) and mean cell hemoglobin concentration (MCHC) (r = −0.254, *P* = 0.007) and a positive correlation with reticulocyte percentage (r = 0.35, *P* < 0.001), bilirubin (r = 0.22, *P* = 0.019), and aspartate transaminase (AST) (r = 0.199, *P* = 0.036). In multivariate regression of the factors determining eGFR, only steady state Hb (β = −0.252, *P* = 0.012) and reticulocyte percentage (β = 0.221, *P* = 0.027) remained independently predictive (R = 0.401, R^2^ = 0.161, F(2,107) = 8.4, *P* < 0.001).

**Table 2:**
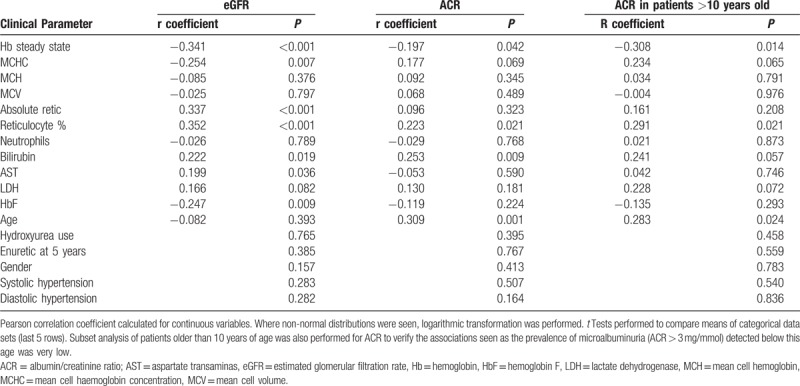
Univariate Analysis of Estimated GFR and Albumin/Creatinine Ratio With Measured Clinical Parameters

In univariate analysis, ACR similarly correlated with low Hb, high reticulocyte percentage and bilirubin, and age (r = −0.197, *P* = 0.042, r = 0.22, *P* = 0.021, r = 0.253, *P* = 0.009, r = 0.308, *P* = 0.001, respectively). In this cross-sectional dataset we found that, with the exception of 1 patient, microalbuminuria was present only in patients older than 10 years of age. This finding is consistent with previous reports.^4,6,21^ To ensure the data collected for children below this age group were not distorting the results, we further analyzed the subset of children over the age of 10 years. In this group of 65 patients, the same associations were found, but with stronger correlation coefficients (data not shown). In multivariate analysis, only age (β = 0.511, *P* = 0.001) and steady state Hb (β = −0.119, *P* = 0.05) were independently significant (R = 0.356, R^2^ = 0.127, F(2,103) = 7.478, *P* = 0.001). There was no correlation with systolic or diastolic hypertension, use of HU or persistence of enuresis beyond 5 years of age with either eGFR (*P* = 0.283, *P* = 0.282, *P* = 0.765, *P* = 0.385, respectively) or ACR (*P* = 0.540, *P* = 0.836, *P* = 0.458, *P* = 0.559, respectively) (Table 2).

**Table 3:**
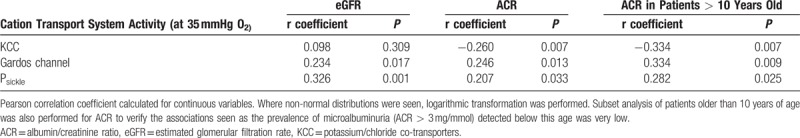
Univariate Analysis of Estimated GFR and Albumin/Creatinine Ratio With Red Cell Cation Transport Systems

**Figure 2:**
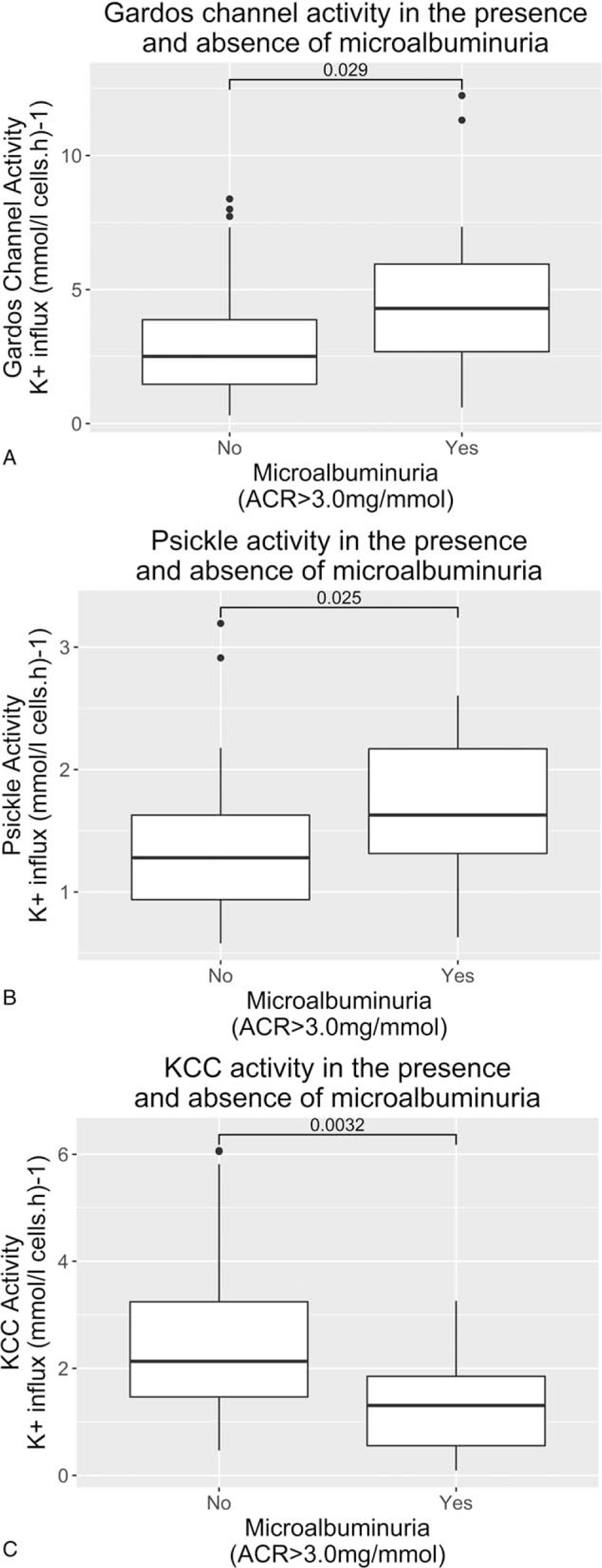
Comparison of red cell transport system activity measurements in the presence or absence of microalbuminuria: (A) Gardos channel, (B) P_sickle_ conductance, (C) KCl co-transporter (KCC).

Alongside routine clinical measurements, red cell cation transport activities were measured. Renal medullary hypoxia is well recognized; studies in rat and renal medullas have reported physiological oxygen tensions of between 15 and 30 mmHg.^22^ We measured transport activities at an oxygen tension of 35 mmHg to assess red cell physiology under similar conditions (Table 3). eGFR showed a modest positive correlation with Gardos channel (r = 0.234, *P* = 0.002) and P_sickle_ (r = 0.326, *P* = 0.005) but no association with KCC activity (r = 0.098, *P* = 0.309). ACR demonstrated similar positive associations with Gardos channel (r = 0.246, *P* = 0.013) and P_sickle_ (r = 0.207, *P* = 0.033) activity, but in contrast, KCC activity was negatively associated with ACR (r = −0.334, *P* = 0.007). Again, looking at the subset of older children, with respect to ACR, all above association remained statistically significant and the correlation coefficients strengthened. Figure 2 demonstrates the variation in cation transport activity between patients with proteinuria, defined as ACR >3 mg/mmol, and those without. Activity levels via both Gardos channel and P_sickle_ were higher in patients with microalbuminuria (*P* = 0.029, *P* = 0.025, respectively), while KCC activity was significantly lower (*P* = 0.003).

When the cation transport variables were added to the previous multivariate regression models, the significant predictors of eGFR were unchanged, namely lower steady state Hb (β = −1.35, *P* = 0.014) and higher reticulocyte percentage (β = 0.1, *P* = 0.026). However, the model for ACR prediction changed. Gardos channel (β = 0.476, *P* < 0.001) and KCC (β = −0.216, *P* = 0.009) activity, alongside age (β = 0.237, *P* = 0.004) were the only independent predictors (R = 0.600, R^2^ = 0.360, F(3,101) = 18.903, *P* < 0.001). Moreover, the R^2^ value has markedly improved to demonstrate a superior predictive model of ACR than that by age and steady state Hb alone.

Correlation of the cation transport activities, at 35 mmHg oxygen tension, with the clinical parameters were assessed (Table 4). Both Gardos channel and P_sickle_ activity showed moderate to strong correlation with markers of hemolysis, namely low Hb (r = −0.414, *P* < 0.001 and r = −0.562 *P* < 0.001), high reticulocyte percent (r = 0.589, *P* < 0.001 and r = 0.676, *P* < 0.001), bilirubin (r = 0.355, *P* < 0.001 and r = 0.384, *P* < 0.001), AST (r = 0.345, *P* < 0.001 and r = 0.373, *P* < 0.001), and lactate dehydrogenase (LDH) (r = 0.299, *P* = 0.001 and r = 0.320, *P* = 0.001) as well as a negative associate with HbF level (r = −0.327, *P* < 0.001 and r = −0.394, *P* < 0.001), but no association with age. Gardos channel also showed correlation with MCHC (r = 0.199, *P* = 0.042). KCC activity showed a different pattern. There was a negative association with steady state Hb (r = −0.245, *P* = 0.010) and MCHC (r = −0.353, *P* < 0.001) but no association with hemolytic markers. There was also a statistically significant negative association with age (r = −0.192, *P* = 0.045) and HbF levels (r = −0.206, *P* = 0.032). Unsurprisingly, given the above associations, P_sickle_ and Gardos channel demonstrated strong correlation with each other (r = 0.74, *P* < 0.001) but KCC did not have statistically significant association with either of the other 2 cation transport mechanisms.

**Table 4:**
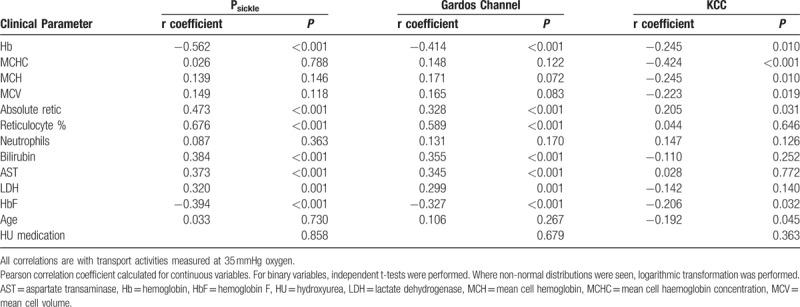
Correlation of Red Cell Cation Transport Systems With Measured Clinical Parameters

## Discussion

This study recruited a large cohort of patients with sickle cell anemia to investigate causes and associations of early sickle nephropathy.

The primary finding of this study is that in multivariate regression analysis, Gardos channel and KCC activity, along with age, are the most significant predictors of ACR, independent of routine clinical measurements. This 3 variable model accounted for 36% of the variability in ACR seen.

Of interest is the divergence of measured activities seen between the 3 cation transport systems. Gardos channel and P_sickle_ show strong concordance with each other (R = 0.74, *P* < 0.001) and both positively correlate with microalbuminuria measurements as our hypothesis would suggest. Conversely, KCC activity shows no concordance with the other 2 cation transport systems and inversely associates with microalbuminuria, that is, increased KCC activity measurement represents a renoprotective state. When multivariate analysis was applied to the channel activities, Gardos and P_sickle_ were clearly co-linear variables, whereas KCC was not predicted by either of the other channel activities suggesting its independence (data not shown). KCC activity was predicted to some extent by patient age. This fall in activity with increasing age is previously observed yet poorly understood.^23^ Some of the association between high KCC activity and low ACR may be related to this phenomenon, although the multivariate analysis suggests that age and KCC independently effect ACR, at least to some extent. The physiology underpinning this divergence of influence of the cation channels is unclear and warrants further investigation. P_sickle_ and Gardos channel are thought to mediate red cell dehydration through different mechanisms and under different conditions to KCC.^10^ P_sickle_ activity leading to increased Gardos channel activity are more directly related to HbS polymerization. KCC, however, with its complex mechanisms of regulation involving multiple conjugate pairs of protein kinases and phosphatases, and intracellular Mg^2+^ levels, is less so.^24^ KCC, a volume-sensitive transport system, is present in normal RBCs, but only active in large, young RBCs contributing to solute loss, red cell shrinkage and maturation from reticulocyte to mature erythrocyte. In SCD, activity is around 50-fold higher, partly due to increased expression, but primarily through abnormal regulation. Activity is also highest in larger, less dense and younger RBCs.^25^ In our study, the negative association of KCC with MCHC seen may reflect this characteristic. Moreover, KCC activity is at its lowest at low oxygen tensions of around 30 mmHg O_2_, such as that seen in the renal medulla^26^ whereas P_sickle_ and Gardos both increase activity with increasing hypoxia.^14^ Thus, KCC activity is unlikely to be a final precipitator of renal insult, whereas both P_sickle_ and Gardos will be far more susceptible to the unique conditions of the renal medulla. Previous studies have demonstrated that increased circulating dense RBCs associate with markers of hemolysis and sickle complications, in particular renal dysfunction.^27,28^ Given KCC activity is recognized to be lower in this subpopulation, an overall net reduction in KCC activity will be observed in a patient with a higher proportion of these dense RBCs. In contrast to KCC activity, Gardos channel activity associates with increased MCHC and both Gardos Channel and P_sickle_ activity associate with markers of hemolysis and both are positively associated with microalbuminuria. Together, this suggests that microalbuminuria is precipitated by an increased population of older denser RBCs in the circulation, driven by Gardos channel and P_sickle_ mediated cation loss. The reduced KCC activity measured possibly reflects the altered constitution of the RBC population as a whole, namely a greater population of small dense RBCs, relative to the large young RBCs. Further studies are clearly required to understand and investigate this interplay better.

The eGFR measurements in our study were significantly higher than that previously reported in the literature. We found the prevalence of hyperfiltration to be 98%, higher than that reported previously of around 75%.^3,29^ We also did not see the previously reported trend of rising GFR in the first decade of life, followed by a gradual fall late in the second decade. Studies comparing measured and calculated methods have demonstrated that eGFR using Schwarz method overestimates the GFR, similarly, the MDRD formula is recognized to be least precise when calculating high values of GFR. These estimation errors may therefore explain the discrepancy. This may also influence the strength of the conclusions drawn from the clinical and cation transport data. Although univariate analysis demonstrated that Gardos channel and P_sickle_ activities, were significantly associated with eGFR, these variables did not influence the final model in multivariate regression. The multivariate model, including steady state Hb and reticulocyte percentage, was poorly predictive of eGFR, only accounting for 16% of the variability of eGFR. A further consideration with regard to GFR is the unknown significance of hyperfiltration in SCN, which may either be a cause of SCN, or a compensatory mechanism in response to early SCN. Reduction in functioning renal nephrons, perhaps due to papillary necrosis, leads remaining nephrons to hypertrophy and increase filtration.^30^ Additionally, the significance of the hyperdynamic blood flow consequent on the chronic anemia should not be overlooked. A third of patients with thalassemia and predominantly those who were not transfused also have hyperfiltration.^31^ Further longitudinal data are required to fully understand the intricacies of this phenomenon in sickle cell disease and a more refined method for accurately estimating GFR from other biochemical markers such as creatinine and cystatin C.

Although the primary objective of this study was to assess the role of cation transport activity with respect to SCN, this was a comparatively large cohort study and it is worth reflecting on the clinical associations seen. The incidence of proteinuria was similar to that previously reported,^5,6^ as was the age of onset being predominantly after the age of 10 years.^4,32^ For comparison, a large cross-sectional study established a rate of 9.5% in healthy children between 6 and 19 years of age,^20^ while another study specific to Nigerian school children reported a rate as high as 33%,^33^ although this study was significantly flawed in that it did not establish basic phenotype data such as SCA status. As has been previously reported, there was a modest association of both eGFR and proteinuria with hemolytic markers. However, contrary to other studies, we did not find any association with neutrophil count.^3,4^ We also did not find an association with hypertension and either eGFR or microalbuminuria which has been previously reported.^3,5^ Blood pressure in our group was adjusted for age, gender, and height and matched to disease-specific reference ranges to identify hypertension; however, as blood pressure was only recorded at 1 visit the readings are difficult to interpret. We also found no associations with the use of HU, suggesting that neither hyperfiltration nor proteinuria are linked to SCA symptoms used to select patients for this therapy, although it is difficult to establish this from our cross-sectional study and impossible to know the effect HU therapy has had on these measures. Previous reports have shown HU administration reduces glomerular hyperfiltration, and also improves microalbuminuria.^28,34,35^ Moreover, the difference in HbF% between the subpopulation of patients on HU was not significantly different to those not prescribed HU. Compliance with this medication is a well-recognized problem, especially in the pediatric population. This study did not take account of such confounders, looking only at whether it was being prescribed to determine status. This possibly also explains why no variation in cation transport activities was seen between patients on HU and those not.

Red cell dehydration is one of the fundamental pathological processes in sickle cell disease, directly influencing HbS polymerization, and contributing to the increased red cell rigidity, which leads to vaso-occlusion, hemolysis and a whole cascade of downstream abnormalities, including tissue infarction, oxidative stress and hypercoagulability. Initial published analysis of the same cohort demonstrated that P_sickle_ and Gardos channel activity, but not KCC, positively correlated with persistence of enuresis beyond the age of 5 years.^36^ Further analysis here has revealed the activity of both KCC and Gardos channels, alongside age, to be the strongest independent predictors of microalbuminuria in our cohort, over and above the clinical measurements recorded. We suggest KCC and the Gardos channel/P_sickle_ conductance systems represent divergent red cell dehydrating mechanisms and may be responsible for different aspects of sickle cell pathophysiology. It would be most interesting to further investigate these cation channels in other cohorts, for example, young adults with SCA, or indeed those with sickle cell trait, in whom nephrotoxicity is beginning to be recognized as a complication. Nonetheless from our current study, the importance of these cation transport systems in sickle organopathy is clear. At least some of the variation in the activity of these transport pathways is likely to be inherited, and may explain why some children and adults with SCA are predisposed toward renal disease. Moreover, as changes in RBC permeability are likely to be detectable before renal damage occurs, these findings suggest a potential prognostic test for SCN to inform patient management, while pharmaceutical modification of Gardos channel, P_sickle_, and KCC activity may be beneficial in preventing progression of SCN, and, potentially, other forms of sickle organopathy.

## References

[1] Brousse V Makani J Rees DC Management of sickle cell disease in the community. *BMJ* 2014;348:g1765. 24613806 10.1136/bmj.g1765 PMC5612384

[2] Elmariah H Garrett ME De Castro LM Factors associated with survival in a contemporary adult sickle cell disease cohort. *Am J Hematol* 2014;89:530–535. 24478166 10.1002/ajh.23683 PMC3988218

[3] Aygun B Mortier NA Smeltzer MP Glomerular hyperfiltration and albuminuria in children with sickle cell anemia. *Pediatr Nephrol* 2011;26:1285–1290. 21559933 10.1007/s00467-011-1857-2 PMC3187922

[4] Wigfall DR Ware RE Burchinal MR Prevalence and clinical correlates of glomerulopathy in children with sickle cell disease. *J Pediatr* 2000;136:749–753. 10839871

[5] Becton LJ Kalpatthi RV Rackoff E Prevalence and clinical correlates of microalbuminuria in children with sickle cell disease. *Pediatr Nephrol* 2010;25:1505–1511. 20505954 10.1007/s00467-010-1536-8

[6] McKie KT Hanevold CD Hernandez C Prevalence, prevention, and treatment of microalbuminuria and proteinuria in children with sickle cell disease. *J Pediatr Hematol Oncol* 2007;29:140–144. 17356390 10.1097/MPH.0b013e3180335081

[7] Powars R Chan S Hiti A Outcome of sickle cell anemia: a 4-decade observational study of 1056 patients. *Medicine (Baltimore)* 2005;84:363–376. 16267411 10.1097/01.md.0000189089.45003.52

[8] Serjeant GR Higgs DR Hambleton IR Elderly survivors with homozygous sickle cell disease. *N Engl J Med* 2007;356:642–643. 17287491 10.1056/NEJMc066547

[9] Eaton WA Hofrichter J Sickle cell hemoglobin polymerization. *Advances in protein chemistry* 1990;40:63–279. 2195851 10.1016/s0065-3233(08)60287-9

[10] Lew VL Bookchin RM Ion transport pathology in the mechanism of sickle cell dehydration. *Physiol Rev* 2005;85:179–200. 15618480 10.1152/physrev.00052.2003

[11] Alvarez O Miller ST Wang WC Effect of hydroxyurea treatment on renal function parameters: results from the multi-center placebo-controlled BABY HUG clinical trial for infants with sickle cell anemia. *Pediatr Blood Cancer* 2012;59:668–674. 22294512 10.1002/pbc.24100 PMC3396762

[12] Levey AS Bosch JP Lewis JB A more accurate method to estimate glomerular filtration rate from serum creatinine: a new prediction equation. Modification of Diet in Renal Disease Study Group. *Annals of internal medicine* 1999;130:461–470. 10075613 10.7326/0003-4819-130-6-199903160-00002

[13] Pegelow CH Colangelo L Steinberg M Natural history of blood pressure in sickle cell disease: risks for stroke and death associated with relative hypertension in sickle cell anemia. *Am J Med* 1997;102:171–177. 9217567 10.1016/s0002-9343(96)00407-x

[14] Hannemann A Rees DC Tewari S Cation homeostasis in red cells from patients with sickle cell disease heterologous for HbS and HbC (HbSC genotype). *EBioMedicine* 2015;2:1669–1676. 26870793 10.1016/j.ebiom.2015.09.026 PMC4740305

[15] Helal I Fick-Brosnahan GM Reed-Gitomer B Glomerular hyperfiltration: definitions, mechanisms and clinical implications. *Nat Rev Nephrol* 2012;8:293–300. 22349487 10.1038/nrneph.2012.19

[16] Cachat F Combescure C Cauderay M A systematic review of glomerular hyperfiltration assessment and definition in the medical literature. *Clin J Am Soc Nephrol* 2015;10:382–389. 25568216 10.2215/CJN.03080314 PMC4348676

[17] Piepsz A Tondeur M Ham H Revisiting normal (51)Cr-ethylenediaminetetraacetic acid clearance values in children. *Eur J Nucl Med Mol Imaging* 2006;33:1477–1482. 16865393 10.1007/s00259-006-0179-2

[18] Chaiken RL Eckert-Norton M Bard M Hyperfiltration in African-American patients with type 2 diabetes. Cross-sectional and longitudinal data. *Diabetes Care* 1998;21:2129–2134. 9839105 10.2337/diacare.21.12.2129

[19] Flynn JT Microalbuminuria in children with primary hypertension. *J Clin Hypertens (Greenwich)* 2016;18:962–965. 27259969 10.1111/jch.12858 PMC8031638

[20] Jones CA Francis ME Eberhardt MS Microalbuminuria in the US population: third National Health and Nutrition Examination Survey. *Am J Kidney Dis* 2002;39:445–459. 11877563 10.1053/ajkd.2002.31388

[21] Dharnidharka VR Dabbagh S Atiyeh B Prevalence of microalbuminuria in children with sickle cell disease. *Pediatr Nephrol* 1998;12:475–478. 9745872 10.1007/s004670050491

[22] O’Connor PM Kett MM Anderson WP Renal medullary tissue oxygenation is dependent on both cortical and medullary blood flow. *Am J Physiol Renal Physiol* 2006;290:F688–F694. 16219913 10.1152/ajprenal.00275.2005

[23] Rees DC Thein SL Osei A The clinical significance of K-Cl cotransport activity in red cells of patients with HbSC disease. *Haematologica* 2015;100:595–600. 25749827 10.3324/haematol.2014.120402 PMC4420208

[24] Gibson JS Ellory JC K+-Cl- Cotransport in Vertebrate Red Cells. In: Bernhardt I Ellory JC, editors. *Red Cell Membrane Transport in Health and Disease* Berlin, Heidelberg: Springer; 2003; 197–220.

[25] Brugnara C Bunn HF Tosteson DC Regulation of erythrocyte cation and water content in sickle cell anemia. *Science* 1986;232:388–390. 3961486 10.1126/science.3961486

[26] Gibson JS Speake PF Ellory JC Differential oxygen sensitivity of the K+-Cl- cotransporter in normal and sickle human red blood cells. *J Physiol* 1998;511 (Pt 1):225–234. 9679176 10.1111/j.1469-7793.1998.225bi.x PMC2231113

[27] Bartolucci P Brugnara C Teixeira-Pinto A Erythrocyte density in sickle cell syndromes is associated with specific clinical manifestations and hemolysis. *Blood* 2012;120:3136–3141. 22919030 10.1182/blood-2012-04-424184

[28] Bartolucci P Habibi A Stehle T Six months of hydroxyurea reduces albuminuria in patients with sickle cell disease. *J Am Soc Nephrol* 2016;27:1847–1853. 26586692 10.1681/ASN.2014111126 PMC4884099

[29] Ware RE Rees RC Sarnaik SA Renal function in infants with sickle cell anemia: baseline data from the BABY HUG trial. *J Pediatr* 2010;156:66.e1–70.e1. 19880138 10.1016/j.jpeds.2009.06.060 PMC4755353

[30] Hostetter TH Olson JL Rennke HG Hyperfiltration in remnant nephrons: a potentially adverse response to renal ablation. *Am Soc Nephrol* 1981;241:F85–F93. 10.1152/ajprenal.1981.241.1.F85 7246778

[31] Quinn CT Johnson VL Kim HY Renal dysfunction in patients with thalassaemia. *Br J Haematol* 2011;153:111–117. 21332704 10.1111/j.1365-2141.2010.08477.x PMC4250090

[32] Alvarez O Montane B Lopez G Early blood transfusions protect against microalbuminuria in children with sickle cell disease. *Pediatr Blood Cancer* 2006;47:71–76. 16261557 10.1002/pbc.20645

[33] Okpere AN Anochie IC Eke FU Prevalence of microalbuminuria among secondary school children. *Afr Health Sci* 2012;12:140–147. 23056019 10.4314/ahs.v12i2.10 PMC3462545

[34] Aygun B Mortier NA Smeltzer MP Hydroxyurea treatment decreases glomerular hyperfiltration in children with sickle cell anemia. *Am J Hematol* 2013;88:116–119. 23255310 10.1002/ajh.23365 PMC4673980

[35] Laurin LP Nachman PH Desai PC Hydroxyurea is associated with lower prevalence of albuminuria in adults with sickle cell disease. *Nephrol Dial Transplant* 2014;29:1211–1218. 24084325 10.1093/ndt/gft295 PMC4038249

[36] Tewari S Rees DC Hannemann A Nocturnal enuresis and K+ transport in red blood cells from patients with sickle cell anemia. *Haematologica* 2016;101:e469–e472. 27587381 10.3324/haematol.2016.149500 PMC5479620

